# Intratumoral *Fusobacterium nucleatum* in Pancreatic Cancer: Current and Future Perspectives

**DOI:** 10.3390/pathogens14010002

**Published:** 2024-12-26

**Authors:** Domenica Lucia D’Antonio, Anna Zenoniani, Samia Umme, Adriano Piattelli, Maria Cristina Curia

**Affiliations:** 1 Department of Medical, Oral and Biotechnological Sciences, “G. d’Annunzio” University of Chieti-Pescara, Via dei Vestini, 66100 Chieti, Italy; domenica.dantonio@unich.it (D.L.D.); anna.zenoniani@studenti.unich.it (A.Z.); ummesamia95@gmail.com (S.U.); 2 Department of Neuroscience, Imaging and Clinical Sciences, “G. d’Annunzio” University of Chieti-Pescara, Via dei Vestini, 66100 Chieti, Italy; 3 School of Dentistry, Saint Camillus International University of Health and Medical Sciences (UniCamillus), 00131 Rome, Italy; apiattelli51@gmail.com; 4 Facultad de Medicina, UCAM Universidad Católica San Antonio de Murcia, 30107 Murcia, Spain

**Keywords:** intratumoral microbiota, dysbiosis, fusobacterium nucleatum, oncobacterium, pancreatic cancer, PDAC, Fap2, FadA, TIGIT, RadD

## Abstract

The intratumoral microbiome plays a significant role in many cancers, such as lung, pancreatic, and colorectal cancer. Pancreatic cancer (PC) is one of the most lethal malignancies and is often diagnosed at advanced stages. *Fusobacterium nucleatum (Fn)*, an anaerobic Gram-negative bacterium primarily residing in the oral cavity, has garnered significant attention for its emerging role in several extra-oral human diseases and, lately, in pancreatic cancer progression and prognosis. It is now recognized as oncobacterium. *Fn* engages in pancreatic tumorigenesis and metastasis through multifaceted mechanisms, including immune response modulation, virulence factors, control of cell proliferation, intestinal metabolite interactions, DNA damage, and epithelial–mesenchymal transition. Additionally, compelling research suggests that *Fn* may exert detrimental effects on cancer treatment outcomes. This paper extends the perspective to pancreatic cancer associated with *Fn*. The central focus is to unravel the oncogenomic changes driven by *Fn in* colonization, initiation, and promotion of pancreatic cancer development. The presence of *Fusobacterium* species can be considered a prognostic marker of PC, and it is also correlated to chemoresistance. Furthermore, this review underscores the clinical research significance of *Fn* as a potential tumor biomarker and therapeutic target, offering a novel outlook on its applicability in cancer detection and prognostic assessment. It is thought that given the role of *Fn* in tumor formation and metastasis processes via its FadA, FapA, Fap2, and RadD, new therapies for tumor treatment targeting *Fn* will be developed.

## 1. Microbiota in Healthy Pancreas

Several studies are being developed on the relationship between the pancreas and host microbiota, consisting of the regulation of immunity and mutual communication [1,2]. Moreover, metabolism, immunity, and nutrition are only some of the examples in which the role of bacteria can be influential [3].

Anatomically, the pancreas is connected to the gastrointestinal tract via the pancreatic duct and can communicate with the liver via the common bile duct. The close communication between the gastrointestinal tract and pancreas induces one to wonder whether the intestinal microbes or an innate microbiota of the pancreas could have an impact on the homeostatic pancreatic processes such as in the intestine. For this reason, more research is directed to the study of the composition of pancreatic microbiota. At first, it was thought that the pancreas was free of bacteria; however, several studies reported the existence of a microbiota both in pathological and healthy conditions [1,4,5].

A potential route for bacterial introduction into the pancreas is translocation from the gut. This explanation is anatomically plausible, and it is conceivable that the bacteria translocate and reach the pancreas from the intestine, going through the mesenteric venous drainage directed to the liver and passing by the pancreas. Commensal bacteria move from the gut to the mesenteric lymph node independently or phagocytized by intestinal immune mononuclear phagocytes CX3CR1hi and captured from the intestinal lumen [6,7]. For this reason, bacteria enter the pancreas from the intestine by the lymphatic drain, although the specifics of the trafficking remain still uncertain [1].

Given the anatomical closeness of the pancreas to the gastrointestinal tract, there is another possible mechanism whereby the microbiota from the esophagus, stomach, duodenum, or biliary tract could penetrate the pancreatic tissue via the pancreatic duct. However, the possibility that the oral cavity or gastrointestinal tract microbiome could reach the pancreas through blood, as well as lymphatic drainage, is not excluded [8].

Under normal conditions, portal blood may contain small amounts of potential pathogens [9]. In cats, *E. coli* can penetrate through the colon’s transmural wall and spread via the bloodstream to the pancreas, especially in those with acute pancreatitis [10]. Bacterial translocation was detectable in blood samples from patients with acute pancreatitis through 16S rDNA sequencing [11]. However, achieving this blood drainage appears complex in the absence of disease.

Bacteria from the oral cavity, such as *Porphyromonas gingivalis*, may be captured by lymphatic vessels during the flow from the oral cavity to the bloodstream, ultimately entering the systemic circulation [12].

In the normal pancreas, a relative increase in the genus *Brevibacterium* and the order of *Chlamydiales* was detected [4]. The existence of bacteria in a healthy pancreas led us to wonder what function could be covered in pancreatic physiology. In the gastrointestinal system, the antimicrobial peptides (AMPs), mainly produced by Paneth cells of the intestine, play a role in innate immunity against bacteria. It is very likely that the pancreas also serves to enhance this innate defense system [13]. The pancreatic AMPs represent ~10% of products of pancreatic juice; the remainder stands for digestive enzymes [14,15]. There is a two-way connection between pancreatic AMPs and gut microbiota; the intestine microbiota has an impact on AMPs of the pancreas to regulate the intrapancreatic immune cells, but also the production of AMPs in the gastrointestinal system through pancreatic liquid could modify the microbiome of the intestine and its immune system [16,17]. The interaction among the gut microbiota metabolites represents a network that can affect some host operations. During digestion, fermentation, and metabolization of protein, microbes generate glycoproteins and fibers from the diet like acetic, propionic, and butyric acid, called short-chain fatty acids (SCFAs). For example, the behavior of SCFAs in the colon could have an impact on the production of glucagon-like peptide-1 (GLP-1), which can regulate the liberation of hormones in the pancreas [18]. Enteric microbes and acetogens like *Blautia hydrogenotrophica*, *Firmicutes*, and *Bacteroidetes* produce acetate across the acetyl-CoA, the lactate, and the succinate pathways, respectively [19,20]. The generation of propionate is also connected to a restricted number of genera of microbes, like *Akkermansia municiphila*, also capable of the mucin degradation. *Firmicutes*, including *Eubacterium rectale*, *Faecalibacterium prausnitzii*, *Ruminococcus bromii*, and *Eubacterium hallii*, can produce butyrate, and these bacteria are capable of carrying out the fermentation of resistant starch [21]. Immunity, metabolism, and intestinal effects are influenced by SCFAs. They activate receptors such as G-protein-coupled receptors (GPCRs) but also free fatty acid receptors 1, 2, and 3 (FFA1, FFA2, FFA3). They are present in several types of tissue and play a role in controlling metabolic processes and immunity [22]. The function of SCFA receptors in the pancreas remains unknown. In mice, there is evidence that the interaction between SCFAs and FFA2 leads to an increase in the production of glucose and a reduction in insulin in plasma [23]. FFA3 might have an inhibitory role in liberation of insulin, and this could be due to the downstream pathway following the link with Gi proteins. The inhibitory effect on the production of insulin from the β cells of the pancreas is clarified via the signal FFA3 linked with Gi proteins, whereas the stimulation in insulin production can be made thanks to the FFA2 activation of Gq/11 signaling [24,25]. SCFAs could also play a role in the preservation of the intestinal epithelium by suppressing host microbes competitively and in the maintenance of intestinal barrier permeability. In this way, they protect from metabolic endotoxemia connected to obesity, leaky gut-derived insulin resistance (IR), and fat swelling [26,27].

## 2. Pancreatic Cancer Intratumoral Microbiota

Ninety-five percent of pancreatic cancer (PC) originates from its exocrine component and is therefore constituted of ductal and acinar cells, and pancreatic ductal adenocarcinoma (PDAC) is the most common class of carcinoma of the pancreas [28]. PDAC frequently arises from pancreatic intraepithelial neoplasms (PanINs). Less than 10% of patients affected by PDAC could reach survival at 5 years [29]. The diagnosis of PDAC is often done in the last stages because there are not often early specific symptoms, and surgery remains the curative treatment [30]. However, most patients do not survive [31]. Adjuvant chemotherapy (gemcitabine with erlotinib or 5-fluorouracil and cisplatin) and radiotherapy are ineffective [32,33,34]. In the USA, PC will become the second leading cause of cancer death by 2030 [35,36]. Different risk factors like cigarette smoking, intake of heavy alcohol, chronic pancreatitis, obesity, food, type 2 diabetes, late age (median 71 years), gender (men more than women), or familiarity with PC are connected to the growth of PC [30,37]. A small percentage (5–10%) is represented by hereditary forms such as familial pancreatic cancer or associated with inherited syndromes like familial adenomatous polyposis, atypical multiple mole melanoma, and Peutz–Jeghers syndrome [38,39]. A better comprehension of tumor formation as well as the option of early diagnosis and supervision of tumor condition is allowed thanks to the study of the cytogenetic, epigenetic, and genetic alterations in pancreatic cancer [40].

Recurrent mutations of oncogenes and tumor suppressors, together with structural and numerical chromosomal anomalies, characterize the complicated karyotype of PC [41]. The mutational spectrum that characterizes PC is represented by several somatic copy alterations (CNAs) and genetic mutations in four genes: the oncogene KRAS and the tumor suppressors TP53, SMAD4, and CDKN2A. This is well reported in The Cancer Genome Atlas (TCGA) [40]. KRAS is mutated in 93% of patients, showing 19% of the G12R allele, 27% of the G12V allele, and 41% of the G12D allele. KRAS activation is the molecular trait of this disease, and it is the first event in pancreatic cancer formation [42]. The rate of mutation of the suppressor genes was 72% for TP53, 32% for SMAD4, and 30% for CDKN2A. However, less common mutations of genes were found, such as BRCA1, BRCAW, ATM, and PALB2, involved in the repair of DNA damage, or oncogenes such as GATA6, GNAS, AKT2, FGFRQ, MYC, BRAF, and MDM2, tumor suppressors such as PTEN or ARID1A, and PBRMQ and MLL31 involved in the changing of chromatin [40].

Through the innovative work of Warren and Marshall, which associated gastric cancer with Helicobacter pylori [43], the study of the role of bacteria in oncogenesis and in the growth of carcinoma has been improved. It has been found that bacterial species have been involved in the onset and progression of a wide range of tumors. Lately, oral bacteria like *Fusobacterium nucleatum* (*Fn*) have been correlated to the development of tumors far from the oral cavity [44,45]. This group of bacteria is now recognized as oncobacteria [46,47,48]. In the last few years, the human microbiota has become a hot topic in biomedical research due to the development of high-throughput methods that have made it possible to detect hundreds of different species of microorganisms in a very short time [49]. In normal conditions, commensal microbiota and human immunity are in a dynamic balance, leading the immune system to react against host bacteria. But when this balance does not work efficiently, a condition of dysbiosis is established, and the microbiota can induce proinflammatory or immunosuppressive responses that can stimulate tumorigenesis [50,51]. This is the case of PDAC, in which it has recently been reported that progression, diagnosis, treatment, chemotherapy resistance, and immunity modulation can be influenced by the intratumoral microbiota, probably originated from intestinal microbiota [52,53].

Tumors arising from non-digestive tract areas, such as breast cancer, are more likely to have an intratumoral microbiome introduced through blood or lymphatic drainage [45,54]. Although there is currently no direct experimental proof proving that the microbiome can reach PC via blood or lymph from microbial-rich areas like the oral cavity or gastrointestinal tract, considerable indirect evidence sustains the plausibility of such a transport route.

While the accurate mechanism remains unclear, microbial staining of various tumors has shown that the intracellular microbiome is present within macrophages [55,56]. Immunohistochemical (IHC) staining of LPS within macrophages could result from the phagocytosis of local microbiota. Macrophages that test positive for IHC LPS indicate that the LPS staining in macrophages may come from bacterial elements that were not fully processed [55,56]. Thus, it is reasonable that the microbiome within a tumor could be moved to the pancreas through macrophage-mediated lymphatic drainage.

While the origin of the intratumoral microbiome in PC remains uncertain, multiple possible origins are likely involved.

**Figure 1:**
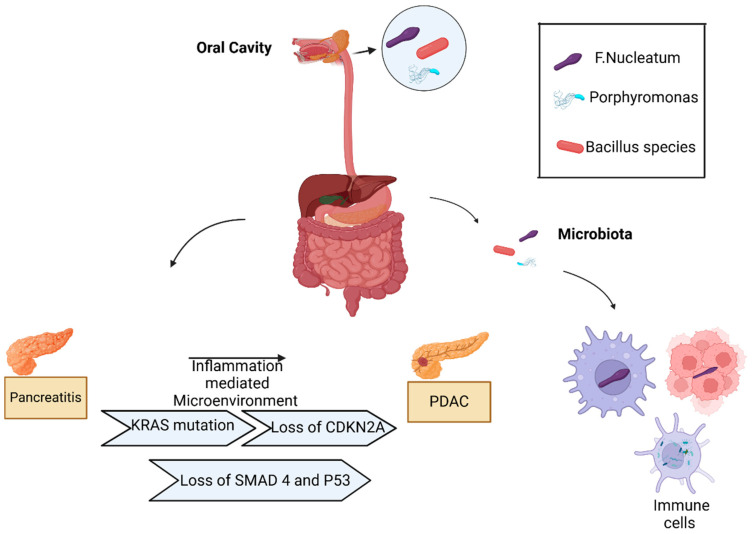
The involvement of intratumoral microbiota in PDAC development. The oral cavity and gut are potential sources of microbiota found in PDAC. Microbiota from the oral cavity and gut can access the pancreas through the pancreatic duct, blood, or lymph. The PDAC intratumoral microbiota is found within tumor cells, immune cells, and the surrounding extracellular environment. Created with BioRender.com.

Riquelme et al. suggested that the gut microbiota can colonize pancreatic tumors specifically. This was demonstrated by comparing the microbiota of tumor tissue, adjacent nontumor, and stool samples from PDAC patients undergoing Whipple surgery. They found that 25% of the intratumoral microbiota was derived from gut microbiota, while there was no trace of it in adjacent healthy tissue [52]. This suggests that the direct transfer of intestinal bacteria and subsequent modifications of its composition lead to pancreatic intratumoral microbiota [52]. In cancer, the immunosuppressive microenvironment discussed above, together with hypoxia and an altered vascular system, are the conditions to allow bacteria to rapidly colonize, grow, and replicate [57]. In particular, the intratumoral pancreatic microbiota may originate from the disruption of the mucosal barrier, from the digestive and cardiovascular systems, and from the normal adjacent tissue (NAT). The different microbiota of the oral cavity, gastrointestinal system, reproductive tract, and skin are in eubiosis with the host. They have not had the opportunity to enter the organism and cause diseases because they are isolated from the host thanks to a mucosal barrier [58]. It has been reported that PDAC intratumoral bacteria can metastasize from the intestinal tract, where the epithelial (mucosal) barrier is disrupted, and enter the pancreas through the pancreatic duct, remodeling the tumor microenvironment (TME) and inducing innate and adaptive immunosuppression (Figure 1). This encourages additional microbial translocations [59]. In pancreatic cancer, the microbiota populates the TME via hematogenic diffusion, and through compromised vessels, it can reach the tumor [60]. During pancreatic cancerogenesis the main origin of gastrointestinal and circulatory system intratumoral microbiota is the oral microbiota. The oral microbiota can disseminate the respiratory and digestive systems thanks to the connection between the oral cavity and these two systems [61]. An elevated rate of pancreatic cancer related to oral dissemination of *Aggregatibacter actino-mycetemcomitans* and *Porphyromonas gingivalis*, together with the presence of antibodies against *Porphyromonas gingivalis*, has been reported during an association study about the incidence of pancreatic cancer and the composition of the microbiota of saliva [62]. In addition, the oral bacteria *Treponema denticola*, *Tannerella forsythia*, and *Prevotella intermedia*, carriers of peptidyl arginine deaminase, can be found in pancreatic cancer as the main cause of the mutation of p53 [63]. The existence of other sources of intratumoral microbiota and normal adjacent tissues (NATs) has been suggested based on a study that investigated seven different types of tumors and their NATs. The authors found a comparable composition of NATs microbiota of breast and lung cancer and their intratumoral microbiota [56]. An explanation for the analogy between intratumoral and NAT microbiota could be due to the origin of NAT from the TME [64].

*H. pylori* was the first pathogenic bacterium found in pancreatic tumor tissue [65]. The DNA of *H. pylori* was found in 75% of pancreatic samples of PDAC patients, in 60% of chronic pancreatitis patients, but in none of the healthy controls [66]; thus, its association with pancreatic cancer was hypothesized. In another study in which the oral microbiota was investigated by 16S rRNA sequencing, *Porphyromonas gingivalis* and *Aggregatibacter actinomycetemcomitans* were associated with a risk of developing PDAC [67]. *Streptococcus* and *Leptotrichina* were also associated with an increased risk of PDAC development compared to healthy controls. A reduced risk of PDAC has been instead related to the presence of *Veillonella* and *Neisseria* in addition to having protective functions. Patients with a high presence of oral *Porphyromonas*, *Fusobacterium*, and *Alloprevotella* tended to usually report bloating. Furthermore, a considerably higher increase in the commensal oral bacteria *Fusobacterium* spp. was detected in PDAC samples in comparison to controls, and its presence was associated with a worse prognosis [68,69]. A large presence of *Prevotella* has been reported in patients presenting with jaundice [70]. Dark-brown urine was found in patients with a high presence of *Veillonella*, whereas patients with a low number of *Neisseria*, *Campylobacter*, and *Alloprevotella* presented with diarrhea and vomiting, respectively. The symptoms mentioned above should lead patients to seek medical care, which could lead to early diagnosis and better prognosis. Through the study of Chung et al., the microbiota of oral cavity, pancreatic, and intestinal tissues was isolated from 52 subject samples [70]. 16S rRNA genes were characterized using high-throughput DNA sequencing. Different taxa of bacteria in samples of oral cavity and intestinal and pancreatic tissue were detected. It was observed that PC patients and healthy controls had different co-abundance patterns, with oral, intestinal, or pancreatic samples from *Fn* subsp. *vincentii* and *Gemella morbillorum* present or absent. These results show that the presence or lack of specific groups of bacteria throughout different positions is related to the development of PC or other diseases of the gastrointestinal system [70]. Different studies comparing healthy and cancerous tissue have shown the presence of *Firmicutes* and *Proteobacteria*, which are the same bacteria present in the healthy intestine [8,56,65]. Contrarily, healthy controls show a higher presence of *Lactobacillus* than in PDAC patients [68]. In pancreatic cancer patients, an increased number of *Selenomonas*, *Enterobacter*, *Klebsiella*, and *Prevotella* was detected both in the pancreas and in stools [56,68,71]. An increment of *Capnocytophaga*, *Citrobacter*, *Haemophilus*, and *Parvimonas* was also reported within the pancreatic TME [56,68]. Intriguingly, unique microbiota have been detected in the fluid of pancreatic cysts [72], with a predominance of the oral *Fn* and *Granulicatella adiances* in the fluid of pancreatic cysts of intraductal papillary mucinous neoplasms (IPMNs) in comparison to non-IPMN pancreatic cystic neoplasia. Considering the development of IPMN in invasive PC, these data suggest the pathogenicity of these bacterial species and underline their possible colonization from the oral cavity [62].

The pathway through which the intratumoral microbiota promotes tumor generation acquired more and more attention. Changes in intratumoral or neighboring microbial communities in cancer patients are referred to as the tumor-associated microbiome [73]. Intratumoral microbiota is able to hamper the defense mechanism of the body related to genetic mutation, and this can lead to the promotion of tumorigenesis of the pancreas [74]. The main hypothesized mechanisms are the damage to DNA due to the secretion of metabolites and the alteration in the tumor immune microenvironment. In pancreatic tumorigenesis, an important role in inducing DNA damage and mutations is exerted by metabolites produced by the microbiome, such as cytolethal distending toxin, colibactin, and *Bacteroides fragilis* toxin [75,76]. Some Gram-negative bacteria, which belong to the ε and g classes of the *Proteobacteria* phylum, generate CDT [77]. This is composed of three protein subunits, CdtA, CdtB, and CdtC, and CdtB is especially connected to DNA damage [78,79]. *Bacteroides fragilis* generates BFT, which can produce an increased number of reactive oxygen species, and through the upregulation of spermine oxidase, it can lead to DNA damage, which could be implicated in the induction of tumorigenesis of the colon [76]. It also has the ability to release PGE2, which is responsible for the inflammatory reaction across the activation of the expression of cyclooxygenase-2, connected to the development of colon cancer [80]. The most marked metabolite secreted by intratumoral microbiota and involved in the DNA damage is colibactin. It is generated by group B2 of *E. coli* strains and has the potential to induce cancerous changes across genomic instability and break DNA double strands [81]. In addition, mutation of arginine in oncogene KRAS and tumor suppressor gene TP53, which are considered the cause of PDAC, are determined by the degradation of arginine by peptidyl–arginine deiminase secreted by oral microbiota in the pancreas [82]. Also, the secretion of SCFAs by the intestinal microbiota has been considered of considerable importance for PC development, progression, and clinical outcomes [83]. These metabolites, including acetate, propionate, and butyrate, are derived from the gut microbiota across the fermentation of nutritional fiber or other supports [84]. An important number of tumor-associated mechanisms like inflammation, cell proliferation, and immune response are regulated by SCFAs, as emerging evidence proposes [85]. In PDAC, prognosis can be influenced by SCFAs and intestinal microbiota through the control of the tumor microenvironment and host immunity [86]. On the other hand, an immunosuppressive microenvironment and worse outcomes are generated by dysbiosis and alterations in SCFAs [86]. The intratumoral microbiota can produce metabolites that can lead to tumor development through inflammatory and immunosuppressive reactions and to the creation of an immunosuppressive microenvironment favorable for tumor progression [3]. It also has the potential to trigger pancreatic tumorigenesis by suppressing immunity through the alteration in myeloid-derived suppressor cells (MDSCs), regulatory T cells (Tregs), and presentation of antigens [73].

## 3. *Fn* Oncobacterium and Its Pathogenic Mechanism in Pancreatic Cancer Development

*Fn* is a non-spore-forming, obligatory anaerobic Gram-negative bacillus that is a member of the genus *Fusobacterium*, which gets its name from its slender form and spindle-like tips on both ends [87]. First identified as an oral pathobiont, *Fn* is known to coaggregate with different types of microorganisms in the oral cavity, influencing the state of periodontal health and disease by playing a crucial role in the formation of dental plaque [88,89,90,91]. The expression of some adhesion proteins, like *Fusobacterium* outer membrane protein A (FapA), *Fusobacterium* autotransporter protein 2 (Fap2), and radiation-sensitive DNA adhesins (RadD), allows residues 68–123 to 68–125 *Fn* to function as a link between early colonizers (e.g., *Streptococcus* species) and later invaders (e.g., *Porphyromonas gingivalis)* [92]. This makes it easier for biofilms to form and stick firmly to the surfaces of teeth. *Fn* directly influences host responses and makes other pathogens more infectious. For this reason, it is significant in periodontitis even though the oral biofilms are present on tooth surfaces in healthy people. In the oral epithelium, *Fn* can specifically stimulate the expression of proinflammatory cytokines like IL-6 and IL-8 as well as the antimicrobial peptide β-defensin 2 [93,94,95]. This type of *Fn*-driven inflammation advances the course of the disease in an oral tumorigenesis model [96,97]. In these pathogenic environments, *Fn* affects the function of immune cells, including myeloid cells, by activating NF-κB, which leads to the production of TNF [98]. *Fn* not only alters these host reactions but also makes *Porphyromonas gingivalis* more invasive, implying that these bacteria work together to avoid immune system destruction and create an environment that is inflammatory and permissive during periodontitis [99,100]. When it infiltrates sterile areas like the root canal, *Fn* functions as an opportunistic pathogen in patients with weakened immune systems [46,89].

*Fn* is the most studied oncobacterium in a variety of cancer types such as colon, breast, oral, pancreatic, esophageal, gastric, and cervical cancer [45,96,101,102,103,104,105,106,107]. Frequently it occurs as a commensal in different sites of the body, particularly the oral cavity [87]. Due to its virulence mechanisms, which include the capacity to cause tumorigenesis and abnormal inflammation, and to its dissemination through the hematogenous route, it has frequently been associated with several extra-oral diseases, including cancers [88,108]. It is logical to assume that *Fn* found in gut tumors may have originated from the oral cavity given the anatomical relationship between the intestinal tract and the oral cavity and the discovery of identical *Fn* strains in both oral and gastrointestinal cancer samples [44]. The ability of *Fn* to survive in acidic environments and move through the gastrointestinal tract is due to another *Fn* protein, the adhesin FadA. It is the primary *Fn* virulence factor, as has been clarified by recent studies [103,109]. FadA is also an invasin [110]. Constant swallowing of bacterial-rich saliva offers a possible route of transmission through the gastrointestinal tract. The increased frequency of *Fn* and FadA in fecal samples from patients with colorectal cancer [111] supports this. Furthermore, post-intravenous injection detection of *Fn* strains in colon cancer tissues raises the possibility of systemic colonization via circulation [112]. Moreover, glycan–lectin interactions are responsible for the localization of *Fn* within tumors. Fap2, a galactose-adhesive hemagglutinin, has been demonstrated to mediate *Fn* colonization through its binding to the host factor Gal-GalNAc, which is overexpressed in tumors [113]. Ovarian, prostate, colorectal, pancreatic, and breast cancers show Gal-GalNAc overexpression [91,114]. At the same time, the *Fn* DNA load in these tumors dramatically increases, suggesting that *Fn* may accumulate in cancers with high Gal-GalNAc levels [45,115]. When taken together, these results suggest a tenable mechanism for *Fn* translocation. Numerous studies have demonstrated the important role that *Fn* plays in the pathogenicity, development, and prognosis of CRC and have offered epidemiological and/or experimental evidence of a significant association between *Fn* and CRCs [103,116]. When compared to controls, *Fn* was found in pancreatic tumor cells at noticeably higher levels, indicating a possible link between it and the development of pancreatic cancer [56].

**Figure 2:**
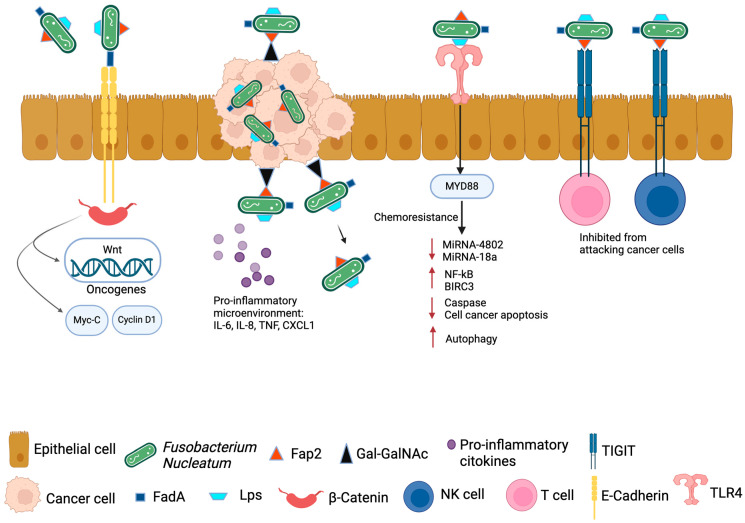
Virulence factors of *Fusobacterium nucleatum. Fn* has three main virulence factors: FadA, LPS, and Fap2. FadA binds E-cadherin, resulting in Wnt/β-catenin signaling that stimulates the transcription of cyclin D and c-Myc, resulting in the development and progression of pancreatic tumors. Fap2 interacts with the TIGIT receptor on NK and T-cells, inhibiting their ability to attack tumor cells. Fap2 also binds to the Gal-GalNac polysaccharide expressed by tumor cells, which localized the *Fn* to tumor cells. *Fn* induces the production of proinflammatory cytokines by tumor cells and immune cells, creating a proinflammatory microenvironment. *Fn* binding of LPS to TRL-4/nuclear factor-kappa B (NF-κB) pathway promotes chemoresistance. It activates the MYD88 innate immune signaling pathway, causing the loss of microRNAs miR-18a and miR-4802, up-regulating autophagy elements, and inhibiting cancer cell apoptosis by up-regulating baculoviral inhibitor of apoptosis protein repeat 3 (BIRC3). Created with BioRender.com. LPS, lipopolysaccharide; Fap2, fusobacterium autotransporter protein 2; TIGIT, T-cell immunoreceptor with Ig and ITIM domains; Ga-GalNac, D-galactose-β (1-3)-N-acetyl-D-galactosamine; TRL-4, toll-like receptor 4.

Intratumoral *Fn* in pancreatic cancer activates oncogenic pathways and regulates the signaling molecules that promote pancreatic tumorigenesis. *Fn* presence and its role as a possible risk factor for pancreatic tumorigenesis have been reported in a study of early cystic precursors in invasive pancreatic cancer by the use of PacBio and qPCR sequencing [117]. FadA facilitates bacterial adhesion to the host mucosal surface and induces damage to endothelial or epithelial cells. In addition, FadA promotes β-catenin signaling and regulates E-cadherin, which increases the expression of Wnt genes, inflammatory genes, transcription factors, and oncogenes [96,118]. Wnt/β-catenin is a signaling pathway that influences stem cell growth, polarization, and self-renewal and controls physiological processes. Through frequent modifications to signaling pathways in the pancreas, Wnt/β-catenin stimulates the transcription of cyclin D and c-Myc, resulting in the development and progression of pancreatic tumors [119,120]. Furthermore, *Fn* triggers toll-like receptor 4 (TLR4) signaling to NF-κB and MyD88, targeting RASA1 and upregulating the expression of miR-4802 and miR-18a, promoting tumorigenesis [121]. Additionally, through the Fap2 adhesin, *Fn* interacts with pancreatic cancer cells, promoting *Fn* infection in pancreatic cancer by causing infected tumor cells to release cytokines such as GM-CSF, CXCL1, IL-8, and MIP-3α, thereby promoting further tumor progression (Figure 2). GM-CSF substantially speeds up the proliferation of pancreatic cancer cells, and CXCL1 is essential for metastasis and chemotherapy resistance in pancreatic cancer. Furthermore, it has been discovered that *Fn* invades healthy pancreatic epithelial cells, promoting tumor cell migration and proliferation [122]. Moreover, it has recently been discovered that *Fn* in pancreatic tumors can affect the development of pancreatic cancer by altering the immune environment surrounding the tumor. To enhance tumorigenesis, *Fn* modifies the tumor immune microenvironment by specifically attracting tumor-infiltrating myeloid cells, including dendritic cells (DC), tumor-associated macrophages (TAMs), MDSC, and CD11b myeloid cells [123]. Inflammation and immune responses are influenced by the chemokine CXCL1, which also serves as an immune cell chemoattractant. It affects tumor migration and proliferation by binding to a particular receptor called CXCR2, which initiates a series of various signaling events. This research has demonstrated that intratumoral *Fn* stimulates tumor growth by increasing pancreatic cancer cells’ autocrine production of CXCL1. To further aid the tumor’s immune evasion, intratumoral *Fn* paracrinely suppresses CD8+ T cells and recruits MDSCs in the tumor microenvironment through the CXCL1/CXCR2 axis [104]. PTEN dysfunction has been reported as an example of immune evasion mechanisms. It has been demonstrated that *Fn* down-regulates PTEN expression by upregulating miR-21 levels, which promotes immune evasion by pancreatic cancer cells [91]. PTEN dysfunctions or mutations result in an immune-suppressive tumor microenvironment characterized by the modulation of M2 macrophages, MDSCs, and Tregs [91]. By interacting with the TIGIT receptor, Fap2 of *Fn* inhibits T cell activation and natural killer (NK) cell cytotoxicity, disrupting the anti-tumor response and generating an immunosuppressive environment [96] (Figure 2).

Intratumoral *Fn* has an impact on pancreatic cancer metastases. Two potential pathways that encourage pancreatic tumor metastasis are disruption of the gut vascular barrier and small extracellular vesicles (sEVs) released by pancreatic cancer cells. Vesicles with a phospholipid bilayer membrane structure that are 150 nm in size are known as sEVs. They can mediate communication between cells and carry proteins, lipids, DNA, and RNA [124]. Intratumoral microbiota-infected pancreatic tumor cells secrete more sEVs by transferring proteins and miRNAs to healthy cells, which encourages the spread of pancreatic cancer [125]. Intratumoral *Fn* can accelerate the development of pancreatic cancer by infecting sEVs carrying miR-92b-3p/27a-3p/1246 in pancreatic tumors, which in turn activates the Wnt/β-catenin pathway [126]. Furthermore, the intratumoral *Fn’*s activation of TLR4 via secreted sEVs encourages the metastasis of pancreatic tumors [127]. Several studies have shown that sEVs mediate communication between cells in distant organs and between pancreatic cancer cells and their surrounding microenvironment, remodel the extracellular matrix, encourage angiogenesis, and create an immunosuppressive environment. This creates a pre-metastatic niche that facilitates pancreatic cancer metastasis [128].

## 4. *Fusobacterium nucleatum* and Pancreatic Cancer Prognosis, Therapy, and Biomarkers

*Fn* could be considered a biomarker for the detection of cancer, as its presence is related to cancer status in CRC patients [103,129]. In our previously published study, the presence of the bacterium in the colon tumor tissue from 36 patients had a statistically significant influence (*p* = 0.016) on staging [97]. Furthermore, an increase in oral *Fn* concentrations was correlated with an increase in colorectal tissue *Fn* quantity. For this reason, *Fn* could be considered a prognostic marker of staging. Furthermore, a cut-off amount of *Fn* in the oral cavity could be considered as one of the identifying markers of PC or at least as a risk factor.

Mitsuhashi and colleagues analyzed 283 patients with PDAC to seek in cancerous tissue samples the presence of *Fn* and to examine the role played by *Fn* in this disease. They found species of *Fusobacterium* in 8.8% of a sample of PC tissue and compared it to median cancer survival in two groups; the life expectancy of the *Fusobacterium* species-positive group decreased considerably (17.2 months versus 32.5; log-rank *p* = 0.021). It was concluded that the presence of *Fusobacterium* species can be considered a prognostic marker of PC [69]. Chemotherapy resistance with a high presence of *Fn* in cancers was noted [105,130,131]. This is due to the interactions of the oncobacterium with the therapeutic factors or to modifications in the immunological milieu of cancer, reducing the effectiveness of these methods. These connections lead to the mitigation of the sensitivity of drug [132]. The study of Michaud et al. was the first report that demonstrated an association of tumor presence of *Fusobacterium* species with the outcome of pancreatic cancer in patients with stories of periodontal disease [133]. Despite the absence of any significant connection, species of *Fusobacterium* were found in cancer of the pancreatic tail (4/18; 22%) more than in body (5/62; 8.0%) or head cancer (16/203; 7.9%) [69]. The reason why there is this important presence of species of *Fusobacterium* in cancer of the pancreatic tail remains unclear. The divergence of circulatory supply between these components of the pancreas could be considered as one likely interpretation. Furthermore, in the cases of *Fusobacterium* species-positive cancer, this bacterium was found in 28% of the adjacent normal tissue, suggesting that it may play a role in carcinogenesis [69]. The results of this study might clarify key concepts of carcinogenesis and develop new diagnostic therapeutic methodologies (i.e., eradication) for pancreatic cancer patients. However, owing to cross-sectional (observational) design and the risk of bias, such as selection criteria, different treatments, and exclusion of cases without available tumor tissue. Mitsuhashi et al. corrected regression analysis results to exclude potential confounding factors, which include disease stage, year of diagnosis, and genetic factors such as CpG island methylator phenotype (CIMP) and miRNA expression. The greater presence of DNA of *Fn* in cancerous tissue, added to worse clinical outcomes, could be explained through its function in promoting alterations connected to mutation of molecular traits in tumors, like high microsatellite instability (MSI) [134]. Furthermore, *Fn* plays a role in the prognosis of metastatic colon cancer patients. A study focused on the analysis of DNA in tissue of metastatic colon cancer patients showed the absence of progress and low survival rates in patients with the presence of *Fn* in tumors and in feces [134,135]. Considering the tumor microbiota of PDAC short-term (STS) and long-term survival (LTS) patients, through a study, Riquelme and collaborators discovered a higher alpha-diversity in LTS patients. Additionally, in LTS patients, an intratumoral microbiota containing *Pseudoxanthomonas*, *Streptomyces*, *Saccharopolyspora* and *Bacillus clausii* was identified. This has been identified as a long-term survival indicator, so it can also be considered a good prognostic marker [52].

Immunotherapy, with a focus on the PD-1/PD-L1 axis, is currently the main objective of tumor therapy [136]. *Fn* can interfere with anti-PD-1 inhibitors’ action. Recent studies have revealed that succinic acid, a derivative by *Fn*, interferes with the GMP-AMP synthase-interferon-β pathway, making the body less sensitive to anti-PD-1 monoclonal antibodies and reducing the effectiveness of the immune system in colorectal cancer [137]. Chemotherapeutic agents such as 5-fluorouracil and oxaliplatin produce their therapeutic effects by disrupting the cell cycle [138]. Interestingly, experimental data highlight *Fn’*s ability to trigger cancer autophagy, which is achieved by selectively inhibiting the expression of miR-18a and miR-4802 through the TLR4 and MYD88 pathways. This affects chemotherapy resistance in colorectal cancer [121]. However, by upregulating the expression of the chloride channel protein ANO1 or the apoptosis inhibitor protein BIRC3, *Fn* can also cause resistance to these medications [91]. Furthermore, research has shown that *Fn* activates NLRP3 in ESCC cells (esophageal squamous cell carcinoma), which increases MDSCs and significantly reduces the therapeutic efficacy of cisplatin chemotherapy [139]. Chemotherapy often causes senescence in cancer cells, which is known as therapy-induced senescence. Chemoresistance can be promoted by senescent cells through the senescence-associated secretory phenotype (SASP). *Fn*, following invasion in senescent ESCC cells and induction of DNA damage, can further activate the DNA damage repair pathway, enhancing the SASP. *Fn* thus encourages the release of SASP induced by chemotherapy, which drives the progression of ESCC and chemoresistance [140]. Finally, *Fn* decreases p53 and E-cadherin expression levels in OSCC, primarily via the Wnt/NFAT pathway, which increases tumor cells’ resistance to cisplatin [141].

The utilization of bacteria in tumor diagnosis and prognosis biomarkers holds significant hopes, but the lack of extensive clinical samples and deeper exploration evidence reduces its potential. At the same time, the future prediction is to deepen the unexplored clinical role of *Fn* using multi-omic techniques [92].

Targeting the intratumoral microbiota could be considered an important potentiality in the treatment of PC, but additional research is required to expedite its clinical translation.

In any case, there are some areas that could be promising. The reshaping of the structure of intratumoral microbiota allowed for defining microbial homeostasis. Intratumoral microbiota in PC could be regulated by antibiotics, probiotics, and fecal microbiota transplantation (FMT). Moreover, mostly in the gastrointestinal tract, diet plays a regulatory role for microbiota [142]. The consumption of vegetables, fruits, soy, and fish is connected to a lower risk of pancreatic cancer; on the contrary, the risk becomes higher with the ingestion of meat, fatty products, and sweets [142]. It would therefore be helpful to create a combination therapy relying on the intratumoral microbiota. Through clinical and preclinical studies, it was shown that such functional disturbance of the intestine barrier (IBFD) and apoptosis of crypts in the intestine are determined using radiotherapy [143]. Despite the presence of only a few studies based on the connection between the intratumoral microbiome in pancreatic cancer patients and radiotherapy, it was shown that the composition of microbiota was changed after radiotherapy treatment, especially the decrease in the variety and number of intestinal bacteria species [144]. Hopeful new treatment formulas, including precision therapy, also joined with learning artificial intelligence (AI), are then represented by the typing of bacteria. Some factors, like meal timing, circadian rhythm, sleep, and exercise, were shown to have a role in the influence of postprandial metabolism and the variety of intestinal microbiota [145], so it should be considered for the elaboration of individual-specific treatments.

**Figure 3:**
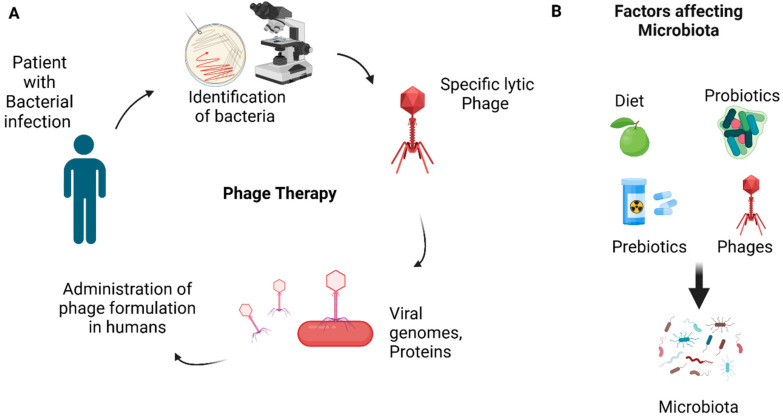
(**A**) Phage therapy with specific lytic phage is shown in the diagram. Specific pathogenic bacterial strain is identified, and a specific strain of bacteriophage (lytic phage) is selected and infected with the bacterial strain. Upon entry of BP into a bacterial cell, it takes over the control of cell machinery, viral genomes and viral proteins are made, the cell undergoes lysis, and new virions are released that can infect other bacterial cells. Safety and efficacy trials on animal models are carried out, and then the formulation is administered to humans after successful clinical trials. (**B**) Some of the important factors affecting microbiota. Created with BioRender.com.

The gastrointestinal microbiota utilizes prebiotics, helpful nutrients for the host, to manipulate the intestinal microenvironment [146]. There are some dietary nutrients that are defined as prebiotics, such as resistant starches that have an impact on the community of microbiota, like through the increased synthesis of SCFAs and protection of DNA from damage [147,148]. Probiotics, prebiotics, and dietary fiber supplementation, meant as specific interventions on intestinal microbiome and SCFA production, may be considered a solution to improve the modulation of the tumor microenvironment and immunotherapy [149,150]. The method by which prebiotics act is antiadhesion against pathogens. To perform this mechanism, prebiotics interact with bacterial receptors mimicking glycoconjugated microvilli in such a way that the pathogens do not attach to the epithelial cells [151,152]. In some tumors, the use of prebiotics is well established (Figure 3). In PC, however, their applications as clinical treatment options need to be better understood [153]. To help in the modulation of dysbiosis and associated tumors, FMT can be utilized due to its significant effectiveness against gastrointestinal pathogens [154]. The substitution of the microbial ecosystem could be representing a possibility to replace the microbiota of patients that host *Fn*. The new microbiota utilizes accurate cocktails of isolates, human-derived or a pool of targeted microorganisms [155].

The end of 2023 (NCT04975217) was designed as the final date for the first phase of trials conducted by the M.D. Anderson Cancer Center to analyze the safety, tolerability, and feasibility of FMT in patients with resectable PDAC [156,157]. The extensive use of FMT encounters a number of difficulties in spite of hopeful progress. The mutable results after FMT are influenced by the donor–recipient affinity, complementary microbiota, own physiology variations, responses of immunity, diet, lifestyle, and genetics [156,157].

The use of probiotics and FMT has been reported to reduce the colonization of *Fn* and to improve the integrity of the gastrointestinal barrier in CRC. In addition to the above treatments, antimicrobial peptides (AMPs) have been presented as aspirant new antimicrobial drugs with significant anti-*Fn* activity [158]. AMPs act as bactericides in mechanically suppressing the *Fn*-induced inflammation. Moreover, AMPs have the advantage of minimal cytotoxicity to colon epithelial cells even at high doses [159].

To measure the *Fn* charge in feces, an approach by search of the fecal occult blood and immunochemical test is proposed as a noninvasive screening, as has been reported for CRC [91]. The search for anti-*Fn* antibodies in saliva and serum by the enzyme-linked immunosorbent assay (ELISA) has been reported for CRC [113]. *Fn* codifies a distinctive amyloid adhesin complex, FadAc, that influences tumor formation. In CRC, anti-FadAc-IgA may represent a biomarker for early diagnosis [160]. The detection of anti-*Fn* antibodies in the blood through ELISA could be a useful PC screening.

The status of mutation of KRAS and TP53, the unstable microsatellite, and the epigenetic dysregulation, which remain undiscussed but relevant [161], can have an impact on the tumoral charge of *Fn* [162].

It has been shown that the majority of isolated clinical cases of *Fn* are sensitive to metronidazole, clindamycin, and some β-lactam antibiotics, except penicillin, to which they are resistant [163]. Another interesting target may be represented by the *Fn* adhesin Fap2 since it promotes the presence of the *Fn* in cancer tissues [112] and affects anti-tumor immunity [164].

Bacteriophages can cut off biofilms [165] and eradicate intracellular bacteria [166]. Phages can modify the immune response during infections of bacteria both in the innate immunity through the release of cytokines and the selection of phagocytes and in specific immunity across the release of antibodies [167]. Kabwe et al. reported that *Klebsiella* and *Fn* are among those microbes whose phages have been detected that could represent a new modality of therapy for PC [168]. Unfortunately, *Porphyromonas gingivalis* bacteria promote their spread and colonization through outer membrane vesicles (OMVs) that allow the systemic spread of the bacteria to colonize distant organs [169,170]. Such a mechanism permits *Porphyromonas gingivalis* to contribute to pancreatic cancer. One lytic bacteriophage against *Fn* has been isolated and characterized. However, no bacteriophage has been found against *Porphyromonas*. As a treatment prospect, the use of bacteriophages to treat antibiotic-resistant pancreatic infections is being considered (Figure 3).

Yamamoto S. et al. [171] investigated the expression of Ki-67, a nuclear marker linked to cell proliferation, in 46 surgical CRC samples to confirm the involvement of *Fn* in the progression of this cancer. High levels of Ki-67 expression correspond to poorer overall survival rates in CRC [172]. *Fn*-positive cancer tissues exhibited a higher Ki-67 index compared to *Fn*-negative tissues, suggesting a significant relationship between *Fn* and cancer cell proliferation.

It is thought that based on the above-mentioned role of *Fn* in tumor formation and metastasis processes, new *Fn*-targeted therapies for tumor treatment will be developed.

## 5. Conclusions

In recent years, the role of the opportunistic oral pathogen has been extensively studied in CRC, while it is still ongoing in PC. Given the anatomic position of the pancreas in the gastrointestinal system, different studies highlighted their attention on the microbiota of the intestine and oral cavity. The mechanisms behind dysbiosis and PC development are not completely clear. There is no doubt that an altered microbiota can lead to oncogenomic changes, and among these bacteria, *Fn* certainly plays an important role. In PC, it has recently been reported that the intratumoral microbiota can influence progression, diagnosis, treatment, chemotherapy resistance, and immunity modulation. An altered oral *Fn* may colonize the pancreas and cause local inflammation by the action of its metabolites, which may lead to carcinogenesis. *Fn* could, therefore, be considered a diagnostic and prognostic biomarker for the detection of cancer. Hopeful new treatments, including precision therapy, oral administration of probiotics, and FMT, are represented by the typing of bacteria. *Fn* is correlated to chemoresistance, and the use of probiotics can improve the effectiveness and the patient’s tolerance to chemotherapy. The utilization of bacteria in tumor diagnosis and prognosis biomarkers holds significant hope, but further studies on a greater sample size are required to expedite its clinical translation.

## Data Availability

No new data were created or analyzed in this study.

## References

[1] Thomas R.M., Gharaibeh R.Z., Gauthier J., Beveridge M., Pope J.L., Guijarro M.V., Yu Q., He Z., Ohland C., Newsome R. (2018). Intestinal microbiota enhances pancreatic carcinogenesis in preclinical models. Carcinogenesis.

[2] Geller L.T., Barzily-Rokni M., Danino T., Jonas O.H., Shental N., Nejman D., Gavert N., Zwang Y., Cooper Z.A., Shee K. (2017). Potential role of intratumor bacteria in mediating tumor resistance to the chemotherapeutic drug gemcitabine. Science.

[3] Wong-Rolle A., Wei H.K., Zhao C., Jin C. (2021). Unexpected guests in the tumor microenvironment: Microbiome in cancer. Protein Cell.

[4] Pushalkar S., Hundeyin M., Daley D., Zambirinis C.P., Kurz E., Mishra A., Mohan N., Aykut B., Usyk M., Torres L.E. (2018). The Pancreatic Cancer Microbiome Promotes Oncogenesis by Induction of Innate and Adaptive Immune Suppression. Cancer Discov..

[5] Sethi V., Kurtom S., Tarique M., Lavania S., Malchiodi Z., Hellmund L., Zhang L., Sharma U., Giri B., Garg B. (2018). Gut Microbiota Promotes Tumor Growth in Mice by Modulating Immune Response. Gastroenterology.

[6] Diehl G.E., Longman R.S., Zhang J.X., Breart B., Galan C., Cuesta A., Schwab S.R., Littman D.R. (2013). Microbiota restricts trafficking of bacteria to mesenteric lymph nodes by CX(3)CR1(hi) cells. Nature.

[7] Bravo-Blas A., Utriainen L., Clay S.L., Kastele V., Cerovic V., Cunningham A.F., Henderson I.R., Wall D.M., Milling S.W.F. (2019). Salmonella enterica Serovar Typhimurium Travels to Mesenteric Lymph Nodes Both with Host Cells and Autonomously. J. Immunol..

[8] Thomas R.M., Jobin C. (2020). Microbiota in pancreatic health and disease: The next frontier in microbiome research. Nat. Rev. Gastroenterol. Hepatol..

[9] Tilg H., Adolph T.E., Trauner M. (2022). Gut-liver axis: Pathophysiological concepts and clinical implications. Cell Metab..

[10] Widdison A.L., Karanjia N.D., Reber H.A. (1994). Routes of spread of pathogens into the pancreas in a feline model of acute pancreatitis. Gut.

[11] Liu J., Huang L., Luo M., Xia X. (2019). Bacterial translocation in acute pancreatitis. Crit. Rev. Microbiol..

[12] Iwai T. (2009). Periodontal bacteremia and various vascular diseases. J. Periodontal Res..

[13] Bevins C.L., Salzman N.H. (2011). Paneth cells, antimicrobial peptides and maintenance of intestinal homeostasis. Nat. Rev. Microbiol..

[14] Medveczky P., Szmola R., Sahin-Toth M. (2009). Proteolytic activation of human pancreatitis-associated protein is required for peptidoglycan binding and bacterial aggregation. Biochem. J..

[15] Doyle C.J., Yancey K., Pitt H.A., Wang M., Bemis K., Yip-Schneider M.T., Sherman S.T., Lillemoe K.D., Goggins M.D., Schmidt C.M. (2012). The proteome of normal pancreatic juice. Pancreas.

[16] Sun J., Furio L., Mecheri R., van der Does A.M., Lundeberg E., Saveanu L., Chen Y., van Endert P., Agerberth B., Diana J. (2015). Pancreatic beta-Cells Limit Autoimmune Diabetes via an Immunoregulatory Antimicrobial Peptide Expressed under the Influence of the Gut Microbiota. Immunity.

[17] Ahuja M., Schwartz D.M., Tandon M., Son A., Zeng M., Swaim W., Eckhaus M., Hoffman V., Cui Y., Xiao B. (2017). Orai1-Mediated Antimicrobial Secretion from Pancreatic Acini Shapes the Gut Microbiome and Regulates Gut Innate Immunity. Cell Metab..

[18] Marroncini G., Naldi L., Martinelli S., Amedei A. (2024). Gut-Liver-Pancreas Axis Crosstalk in Health and Disease: From the Role of Microbial Metabolites to Innovative Microbiota Manipulating Strategies. Biomedicines.

[19] Louis P., Hold G.L., Flint H.J. (2014). The gut microbiota, bacterial metabolites and colorectal cancer. Nat. Rev. Microbiol..

[20] Reichardt N., Duncan S.H., Young P., Belenguer A., McWilliam Leitch C., Scott K.P., Flint H.J., Louis P. (2014). Phylogenetic distribution of three pathways for propionate production within the human gut microbiota. ISME J..

[21] Morrison D.J., Preston T. (2016). Formation of short chain fatty acids by the gut microbiota and their impact on human metabolism. Gut Microbes.

[22] Kimura I., Ichimura A., Ohue-Kitano R., Igarashi M. (2020). Free Fatty Acid Receptors in Health and Disease. Physiol. Rev..

[23] Kimura I., Miyamoto J., Ohue-Kitano R., Watanabe K., Yamada T., Onuki M., Aoki R., Isobe Y., Kashihara D., Inoue D. (2020). Maternal gut microbiota in pregnancy influences offspring metabolic phenotype in mice. Science.

[24] Bolognini D., Moss C.E., Nilsson K., Petersson A.U., Donnelly I., Sergeev E., Konig G.M., Kostenis E., Kurowska-Stolarska M., Miller A. (2016). A Novel Allosteric Activator of Free Fatty Acid 2 Receptor Displays Unique Gi-functional Bias. J. Biol. Chem..

[25] Priyadarshini M., Villa S.R., Fuller M., Wicksteed B., Mackay C.R., Alquier T., Poitout V., Mancebo H., Mirmira R.G., Gilchrist A. (2015). An Acetate-Specific GPCR, FFAR2, Regulates Insulin Secretion. Mol. Endocrinol..

[26] Cani P.D., Amar J., Iglesias M.A., Poggi M., Knauf C., Bastelica D., Neyrinck A.M., Fava F., Tuohy K.M., Chabo C. (2007). Metabolic endotoxemia initiates obesity and insulin resistance. Diabetes.

[27] Riviere A., Selak M., Lantin D., Leroy F., De Vuyst L. (2016). Bifidobacteria and Butyrate-Producing Colon Bacteria: Importance and Strategies for Their Stimulation in the Human Gut. Front. Microbiol..

[28] Pishvaian M.J., Brody J.R. (2017). Therapeutic Implications of Molecular Subtyping for Pancreatic Cancer. Oncology (Williston Park).

[29] Park W., Chawla A., O’Reilly E.M. (2021). Pancreatic Cancer: A Review. JAMA.

[30] Mizrahi J.D., Surana R., Valle J.W., Shroff R.T. (2020). Pancreatic cancer. Lancet.

[31] Hartwig W., Werner J., Jager D., Debus J., Buchler M.W. (2013). Improvement of surgical results for pancreatic cancer. Lancet Oncol..

[32] Moore M.J. (2005). Brief communication: A new combination in the treatment of advanced pancreatic cancer. Semin. Oncol..

[33] Dahan L., Bonnetain F., Ychou M., Mitry E., Gasmi M., Raoul J.L., Cattan S., Phelip J.M., Hammel P., Chauffert B. (2010). Combination 5-fluorouracil, folinic acid and cisplatin (LV5FU2-CDDP) followed by gemcitabine or the reverse sequence in metastatic pancreatic cancer: Final results of a randomised strategic phase III trial (FFCD 0301). Gut.

[34] Hammel P., Huguet F., van Laethem J.L., Goldstein D., Glimelius B., Artru P., Borbath I., Bouche O., Shannon J., Andre T. (2016). Effect of Chemoradiotherapy vs Chemotherapy on Survival in Patients With Locally Advanced Pancreatic Cancer Controlled After 4 Months of Gemcitabine With or Without Erlotinib: The LAP07 Randomized Clinical Trial. JAMA.

[35] Carioli G., Malvezzi M., Bertuccio P., Boffetta P., Levi F., La Vecchia C., Negri E. (2021). European cancer mortality predictions for the year 2021 with focus on pancreatic and female lung cancer. Ann. Oncol..

[36] Rahib L., Smith B.D., Aizenberg R., Rosenzweig A.B., Fleshman J.M., Matrisian L.M. (2014). Projecting cancer incidence and deaths to 2030: The unexpected burden of thyroid, liver, and pancreas cancers in the United States. Cancer Res..

[37] Klein A.P. (2021). Pancreatic cancer epidemiology: Understanding the role of lifestyle and inherited risk factors. Nat. Rev. Gastroenterol. Hepatol..

[38] Solomon S., Das S., Brand R., Whitcomb D.C. (2012). Inherited pancreatic cancer syndromes. Cancer J..

[39] Zhou B., Xu J.W., Cheng Y.G., Gao J.Y., Hu S.Y., Wang L., Zhan H.X. (2017). Early detection of pancreatic cancer: Where are we now and where are we going?. Int. J. Cancer.

[40] The Cancer Genome Atlas Research Network (2017). Integrated Genomic Characterization of Pancreatic Ductal Adenocarcinoma. Cancer Cell.

[41] Heim S., Mitelman F. (2015). Cancer Cytogenetics.

[42] Pfeffer U. (2013). Cancer Genomics: Molecular Classification, Prognosis and Response Prediction.

[43] Marshall B.J., Warren J.R. (1984). Unidentified curved bacilli in the stomach of patients with gastritis and peptic ulceration. Lancet.

[44] Abed J., Maalouf N., Manson A.L., Earl A.M., Parhi L., Emgard J.E.M., Klutstein M., Tayeb S., Almogy G., Atlan K.A. (2020). Colon Cancer-Associated Fusobacterium nucleatum May Originate From the Oral Cavity and Reach Colon Tumors via the Circulatory System. Front Cell Infect. Microbiol..

[45] Parhi L., Alon-Maimon T., Sol A., Nejman D., Shhadeh A., Fainsod-Levi T., Yajuk O., Isaacson B., Abed J., Maalouf N. (2020). Breast cancer colonization by Fusobacterium nucleatum accelerates tumor growth and metastatic progression. Nat. Commun..

[46] Brennan C.A., Garrett W.S. (2019). Fusobacterium nucleatum-symbiont, opportunist and oncobacterium. Nat. Rev. Microbiol..

[47] Kabwe M., Meehan-Andrews T., Ku H., Petrovski S., Batinovic S., Chan H.T., Tucci J. (2021). Lytic Bacteriophage EFA1 Modulates HCT116 Colon Cancer Cell Growth and Upregulates ROS Production in an Enterococcus faecalis Co-culture System. Front. Microbiol..

[48] Rai A.K., Panda M., Das A.K., Rahman T., Das R., Das K., Sarma A., Kataki A.C., Chattopadhyay I. (2021). Dysbiosis of salivary microbiome and cytokines influence oral squamous cell carcinoma through inflammation. Arch. Microbiol..

[49] NIH Human Microbiome Portfolio Analysis Team (2019). A review of 10 years of human microbiome research activities at the US National Institutes of Health, Fiscal Years 2007–2016. Microbiome.

[50] Garrett W.S. (2015). Cancer and the microbiota. Science.

[51] Belkaid Y., Hand T.W. (2014). Role of the microbiota in immunity and inflammation. Cell.

[52] Riquelme E., Zhang Y., Zhang L., Montiel M., Zoltan M., Dong W., Quesada P., Sahin I., Chandra V., San Lucas A. (2019). Tumor Microbiome Diversity and Composition Influence Pancreatic Cancer Outcomes. Cell.

[53] Alam A., Levanduski E., Denz P., Villavicencio H.S., Bhatta M., Alhorebi L., Zhang Y., Gomez E.C., Morreale B., Senchanthisai S. (2022). Fungal mycobiome drives IL-33 secretion and type 2 immunity in pancreatic cancer. Cancer Cell.

[54] Fu A., Yao B., Dong T., Chen Y., Yao J., Liu Y., Li H., Bai H., Liu X., Zhang Y. (2022). Tumor-resident intracellular microbiota promotes metastatic colonization in breast cancer. Cell.

[55] Narunsky-Haziza L., Sepich-Poore G.D., Livyatan I., Asraf O., Martino C., Nejman D., Gavert N., Stajich J.E., Amit G., Gonzalez A. (2022). Pan-cancer analyses reveal cancer-type-specific fungal ecologies and bacteriome interactions. Cell.

[56] Nejman D., Livyatan I., Fuks G., Gavert N., Zwang Y., Geller L.T., Rotter-Maskowitz A., Weiser R., Mallel G., Gigi E. (2020). The human tumor microbiome is composed of tumor type-specific intracellular bacteria. Science.

[57] Walker S.P., Tangney M., Claesson M.J. (2020). Sequence-Based Characterization of Intratumoral Bacteria—A Guide to Best Practice. Front Oncol..

[58] Gonzalez-Sanchez P., DeNicola G.M. (2021). The microbiome(s) and cancer: Know thy neighbor(s). J. Pathol..

[59] Leinwand J., Miller G. (2020). Regulation and modulation of antitumor immunity in pancreatic cancer. Nat. Immunol..

[60] Shi Y., Zheng W., Yang K., Harris K.G., Ni K., Xue L., Lin W., Chang E.B., Weichselbaum R.R., Fu Y.X. (2020). Intratumoral accumulation of gut microbiota facilitates CD47-based immunotherapy via STING signaling. J. Exp. Med..

[61] Ahn J., Chen C.Y., Hayes R.B. (2012). Oral microbiome and oral and gastrointestinal cancer risk. Cancer Causes Control..

[62] Gaiser R.A., Halimi A., Alkharaan H., Lu L., Davanian H., Healy K., Hugerth L.W., Ateeb Z., Valente R., Fernandez Moro C. (2019). Enrichment of oral microbiota in early cystic precursors to invasive pancreatic cancer. Gut.

[63] Ogrendik M. (2015). Oral bacteria in pancreatic cancer: Mutagenesis of the p53 tumour suppressor gene. Int. J. Clin. Exp. Pathol..

[64] Xie Y., Xie F., Zhou X., Zhang L., Yang B., Huang J., Wang F., Yan H., Zeng L., Zhang L. (2022). Microbiota in Tumors: From Understanding to Application. Adv. Sci..

[65] Bao Y., Spiegelman D., Li R., Giovannucci E., Fuchs C.S., Michaud D.S. (2010). History of peptic ulcer disease and pancreatic cancer risk in men. Gastroenterology.

[66] Nilsson H.O., Stenram U., Ihse I., Wadstrom T. (2006). Helicobacter species ribosomal DNA in the pancreas, stomach and duodenum of pancreatic cancer patients. World J. Gastroenterol..

[67] Fan X., Alekseyenko A.V., Wu J., Peters B.A., Jacobs E.J., Gapstur S.M., Purdue M.P., Abnet C.C., Stolzenberg-Solomon R., Miller G. (2018). Human oral microbiome and prospective risk for pancreatic cancer: A population-based nested case-control study. Gut.

[68] Del Castillo E., Meier R., Chung M., Koestler D.C., Chen T., Paster B.J., Charpentier K.P., Kelsey K.T., Izard J., Michaud D.S. (2019). The Microbiomes of Pancreatic and Duodenum Tissue Overlap and Are Highly Subject Specific but Differ between Pancreatic Cancer and Noncancer Subjects. Cancer Epidemiol. Biomark. Prev..

[69] Mitsuhashi K., Nosho K., Sukawa Y., Matsunaga Y., Ito M., Kurihara H., Kanno S., Igarashi H., Naito T., Adachi Y. (2015). Association of Fusobacterium species in pancreatic cancer tissues with molecular features and prognosis. Oncotarget.

[70] Chung M., Zhao N., Meier R., Koestler D.C., Wu G., de Castillo E., Paster B.J., Charpentier K., Izard J., Kelsey K.T. (2021). Comparisons of oral, intestinal, and pancreatic bacterial microbiomes in patients with pancreatic cancer and other gastrointestinal diseases. J. Oral. Microbiol..

[71] Ren Z., Jiang J., Xie H., Li A., Lu H., Xu S., Zhou L., Zhang H., Cui G., Chen X. (2017). Gut microbial profile analysis by MiSeq sequencing of pancreatic carcinoma patients in China. Oncotarget.

[72] Li S., Fuhler G.M., Bn N., Jose T., Bruno M.J., Peppelenbosch M.P., Konstantinov S.R. (2017). Pancreatic cyst fluid harbors a unique microbiome. Microbiome.

[73] Oliva M., Mulet-Margalef N., Ochoa-De-Olza M., Napoli S., Mas J., Laquente B., Alemany L., Duell E.J., Nuciforo P., Moreno V. (2021). Tumor-Associated Microbiome: Where Do We Stand?. Int. J. Mol. Sci..

[74] Scott A.J., Alexander J.L., Merrifield C.A., Cunningham D., Jobin C., Brown R., Alverdy J., O’Keefe S.J., Gaskins H.R., Teare J. (2019). International Cancer Microbiome Consortium consensus statement on the role of the human microbiome in carcinogenesis. Gut.

[75] Barrett M., Hand C.K., Shanahan F., Murphy T., O’Toole P.W. (2020). Mutagenesis by Microbe: The Role of the Microbiota in Shaping the Cancer Genome. Trends Cancer.

[76] Goodwin A.C., Destefano Shields C.E., Wu S., Huso D.L., Wu X., Murray-Stewart T.R., Hacker-Prietz A., Rabizadeh S., Woster P.M., Sears C.L. (2011). Polyamine catabolism contributes to enterotoxigenic Bacteroides fragilis-induced colon tumorigenesis. Proc. Natl. Acad. Sci. USA.

[77] Jinadasa R.N., Bloom S.E., Weiss R.S., Duhamel G.E. (2011). Cytolethal distending toxin: A conserved bacterial genotoxin that blocks cell cycle progression, leading to apoptosis of a broad range of mammalian cell lineages. Microbiology.

[78] Lara-Tejero M., Galan J.E. (2001). CdtA, CdtB, and CdtC form a tripartite complex that is required for cytolethal distending toxin activity. Infect. Immun..

[79] Frisan T., Cortes-Bratti X., Chaves-Olarte E., Stenerlow B., Thelestam M. (2003). The Haemophilus ducreyi cytolethal distending toxin induces DNA double-strand breaks and promotes ATM-dependent activation of RhoA. Cell Microbiol..

[80] Cheng W.T., Kantilal H.K., Davamani F. (2020). The Mechanism of Bacteroides fragilis Toxin Contributes to Colon Cancer Formation. Malays. J. Med. Sci..

[81] Arthur J.C., Gharaibeh R.Z., Muhlbauer M., Perez-Chanona E., Uronis J.M., McCafferty J., Fodor A.A., Jobin C. (2014). Microbial genomic analysis reveals the essential role of inflammation in bacteria-induced colorectal cancer. Nat. Commun..

[82] Tijeras-Raballand A., Hilmi M., Astorgues-Xerri L., Nicolle R., Bieche I., Neuzillet C. (2021). Microbiome and pancreatic ductal adenocarcinoma. Clin. Res. Hepatol. Gastroenterol..

[83] Nouri Z., Choi S.W., Choi I.J., Ryu K.W., Woo S.M., Park S.J., Lee W.J., Choi W., Jung Y.S., Myung S.K. (2023). Exploring Connections between Oral Microbiota, Short-Chain Fatty Acids, and Specific Cancer Types: A Study of Oral Cancer, Head and Neck Cancer, Pancreatic Cancer, and Gastric Cancer. Cancers.

[84] Cummings J.H., Pomare E.W., Branch W.J., Naylor C.P., Macfarlane G.T. (1987). Short chain fatty acids in human large intestine, portal, hepatic and venous blood. Gut.

[85] Al-Qadami G.H., Secombe K.R., Subramaniam C.B., Wardill H.R., Bowen J.M. (2022). Gut Microbiota-Derived Short-Chain Fatty Acids: Impact on Cancer Treatment Response and Toxicities. Microorganisms.

[86] Temel H.Y., Kaymak O., Kaplan S., Bahcivanci B., Gkoutos G.V., Acharjee A. (2023). Role of microbiota and microbiota-derived short-chain fatty acids in PDAC. Cancer Med..

[87] Chen Y., Huang Z., Tang Z., Huang Y., Huang M., Liu H., Ziebolz D., Schmalz G., Jia B., Zhao J. (2022). More Than Just a Periodontal Pathogen—The Research Progress on Fusobacterium nucleatum. Front Cell Infect. Microbiol..

[88] Fan Z., Tang P., Li C., Yang Q., Xu Y., Su C., Li L. (2023). Fusobacterium nucleatum and its associated systemic diseases: Epidemiologic studies and possible mechanisms. J. Oral. Microbiol..

[89] Chen Y., Shi T., Li Y., Huang L., Yin D. (2022). Fusobacterium nucleatum: The Opportunistic Pathogen of Periodontal and Peri-Implant Diseases. Front Microbiol..

[90] Britton T.A., Wu C., Chen Y.W., Franklin D., Chen Y., Camacho M.I., Luong T.T., Das A., Ton-That H. (2024). The respiratory enzyme complex Rnf is vital for metabolic adaptation and virulence in Fusobacterium nucleatum. mBio.

[91] Alon-Maimon T., Mandelboim O., Bachrach G. (2022). Fusobacterium nucleatum and cancer. Periodontology 2000.

[92] Ye C., Liu X., Liu Z., Pan C., Zhang X., Zhao Z., Sun H. (2024). Fusobacterium nucleatum in tumors: From tumorigenesis to tumor metastasis and tumor resistance. Cancer Biol. Ther..

[93] Krisanaprakornkit S., Kimball J.R., Weinberg A., Darveau R.P., Bainbridge B.W., Dale B.A. (2000). Inducible expression of human beta-defensin 2 by Fusobacterium nucleatum in oral epithelial cells: Multiple signaling pathways and role of commensal bacteria in innate immunity and the epithelial barrier. Infect. Immun..

[94] Ahn S.H., Chun S., Park C., Lee J.H., Lee S.W., Lee T.H. (2017). Transcriptome profiling analysis of senescent gingival fibroblasts in response to Fusobacterium nucleatum infection. PLoS ONE.

[95] Bhattacharyya S., Ghosh S.K., Shokeen B., Eapan B., Lux R., Kiselar J., Nithianantham S., Young A., Pandiyan P., McCormick T.S. (2016). FAD-I, a Fusobacterium nucleatum Cell Wall-Associated Diacylated Lipoprotein That Mediates Human Beta Defensin 2 Induction through Toll-Like Receptor-1/2 (TLR-1/2) and TLR-2/6. Infect. Immun..

[96] Pignatelli P., Nuccio F., Piattelli A., Curia M.C. (2023). The Role of Fusobacterium nucleatum in Oral and Colorectal Carcinogenesis. Microorganisms.

[97] Binder Gallimidi A., Fischman S., Revach B., Bulvik R., Maliutina A., Rubinstein A.M., Nussbaum G., Elkin M. (2015). Periodontal pathogens Porphyromonas gingivalis and Fusobacterium nucleatum promote tumor progression in an oral-specific chemical carcinogenesis model. Oncotarget.

[98] Park S.R., Kim D.J., Han S.H., Kang M.J., Lee J.Y., Jeong Y.J., Lee S.J., Kim T.H., Ahn S.G., Yoon J.H. (2014). Diverse Toll-like receptors mediate cytokine production by Fusobacterium nucleatum and Aggregatibacter actinomycetemcomitans in macrophages. Infect. Immun..

[99] Saito A., Kokubu E., Inagaki S., Imamura K., Kita D., Lamont R.J., Ishihara K. (2012). Porphyromonas gingivalis entry into gingival epithelial cells modulated by Fusobacterium nucleatum is dependent on lipid rafts. Microb. Pathog..

[100] Metzger Z., Lin Y.Y., Dimeo F., Ambrose W.W., Trope M., Arnold R.R. (2009). Synergistic pathogenicity of Porphyromonas gingivalis and Fusobacterium nucleatum in the mouse subcutaneous chamber model. J. Endod..

[101] Slade D.J. (2021). New Roles for Fusobacterium nucleatum in Cancer: Target the Bacteria, Host, or Both?. Trends Cancer.

[102] D’Antonio D.L., Marchetti S., Pignatelli P., Piattelli A., Curia M.C. (2022). The Oncobiome in Gastroenteric and Genitourinary Cancers. Int. J. Mol. Sci..

[103] Pignatelli P., Iezzi L., Pennese M., Raimondi P., Cichella A., Bondi D., Grande R., Cotellese R., Di Bartolomeo N., Innocenti P. (2021). The Potential of Colonic Tumor Tissue Fusobacterium nucleatum to Predict Staging and Its Interplay with Oral Abundance in Colon Cancer Patients. Cancers.

[104] Hayashi M., Ikenaga N., Nakata K., Luo H., Zhong P., Date S., Oyama K., Higashijima N., Kubo A., Iwamoto C. (2023). Intratumor Fusobacterium nucleatum promotes the progression of pancreatic cancer via the CXCL1-CXCR2 axis. Cancer Sci..

[105] Yamamura K., Baba Y., Nakagawa S., Mima K., Miyake K., Nakamura K., Sawayama H., Kinoshita K., Ishimoto T., Iwatsuki M. (2016). Human Microbiome Fusobacterium Nucleatum in Esophageal Cancer Tissue Is Associated with Prognosis. Clin. Cancer Res..

[106] Chen W.D., Zhang X., Zhang M.J., Zhang Y.P., Shang Z.Q., Xin Y.W., Zhang Y. (2022). Salivary Fusobacterium nucleatum serves as a potential diagnostic biomarker for gastric cancer. World J. Gastroenterol..

[107] Audirac-Chalifour A., Torres-Poveda K., Bahena-Roman M., Tellez-Sosa J., Martinez-Barnetche J., Cortina-Ceballos B., Lopez-Estrada G., Delgado-Romero K., Burguete-Garcia A.I., Cantu D. (2016). Cervical Microbiome and Cytokine Profile at Various Stages of Cervical Cancer: A Pilot Study. PLoS ONE.

[108] Stokowa-Soltys K., Wojtkowiak K., Jagiello K. (2021). Fusobacterium nucleatum-Friend or foe?. J. Inorg. Biochem..

[109] Meng Q., Gao Q., Mehrazarin S., Tangwanichgapong K., Wang Y., Huang Y., Pan Y., Robinson S., Liu Z., Zangiabadi A. (2021). Fusobacterium nucleatum secretes amyloid-like FadA to enhance pathogenicity. EMBO Rep..

[110] Xu M., Yamada M., Li M., Liu H., Chen S.G., Han Y.W. (2007). FadA from Fusobacterium nucleatum utilizes both secreted and nonsecreted forms for functional oligomerization for attachment and invasion of host cells. J. Biol. Chem..

[111] Wirbel J., Pyl P.T., Kartal E., Zych K., Kashani A., Milanese A., Fleck J.S., Voigt A.Y., Palleja A., Ponnudurai R. (2019). Meta-analysis of fecal metagenomes reveals global microbial signatures that are specific for colorectal cancer. Nat. Med..

[112] Abed J., Emgard J.E., Zamir G., Faroja M., Almogy G., Grenov A., Sol A., Naor R., Pikarsky E., Atlan K.A. (2016). Fap2 Mediates Fusobacterium nucleatum Colorectal Adenocarcinoma Enrichment by Binding to Tumor-Expressed Gal-GalNAc. Cell Host Microbe..

[113] Wang N., Fang J.Y. (2023). Fusobacterium nucleatum, a key pathogenic factor and microbial biomarker for colorectal cancer. Trends Microbiol..

[114] Abed J., Maalouf N., Parhi L., Chaushu S., Mandelboim O., Bachrach G. (2017). Tumor Targeting by Fusobacterium nucleatum: A Pilot Study and Future Perspectives. Front Cell Infect. Microbiol..

[115] Castano-Rodriguez N., Goh K.L., Fock K.M., Mitchell H.M., Kaakoush N.O. (2017). Dysbiosis of the microbiome in gastric carcinogenesis. Sci. Rep..

[116] Sun C.H., Li B.B., Wang B., Zhao J., Zhang X.Y., Li T.T., Li W.B., Tang D., Qiu M.J., Wang X.C. (2019). The role of Fusobacterium nucleatum in colorectal cancer: From carcinogenesis to clinical management. Chronic. Dis. Transl. Med..

[117] Gholizadeh P., Eslami H., Kafil H.S. (2017). Carcinogenesis mechanisms of Fusobacterium nucleatum. Biomed. Pharmacother.

[118] Rubinstein M.R., Baik J.E., Lagana S.M., Han R.P., Raab W.J., Sahoo D., Dalerba P., Wang T.C., Han Y.W. (2019). Fusobacterium nucleatum promotes colorectal cancer by inducing Wnt/beta-catenin modulator Annexin A1. EMBO Rep..

[119] Clevers H., Nusse R. (2012). Wnt/beta-catenin signaling and disease. Cell.

[120] Shang S., Hua F., Hu Z.W. (2017). The regulation of beta-catenin activity and function in cancer: Therapeutic opportunities. Oncotarget.

[121] Yu T., Guo F., Yu Y., Sun T., Ma D., Han J., Qian Y., Kryczek I., Sun D., Nagarsheth N. (2017). Fusobacterium nucleatum Promotes Chemoresistance to Colorectal Cancer by Modulating Autophagy. Cell.

[122] Udayasuryan B., Ahmad R.N., Nguyen T.T.D., Umana A., Monet Roberts L., Sobol P., Jones S.D., Munson J.M., Slade D.J., Verbridge S.S. (2022). Fusobacterium nucleatum induces proliferation and migration in pancreatic cancer cells through host autocrine and paracrine signaling. Sci. Signal.

[123] Borowsky J., Haruki K., Lau M.C., Dias Costa A., Vayrynen J.P., Ugai T., Arima K., da Silva A., Felt K.D., Zhao M. (2021). Association of Fusobacterium nucleatum with Specific T-cell Subsets in the Colorectal Carcinoma Microenvironment. Clin. Cancer Res..

[124] Frydrychowicz M., Kolecka-Bednarczyk A., Madejczyk M., Yasar S., Dworacki G. (2015). Exosomes-structure, biogenesis and biological role in non-small-cell lung cancer. Scand J. Immunol..

[125] McAllister F., Khan M.A.W., Helmink B., Wargo J.A. (2019). The Tumor Microbiome in Pancreatic Cancer: Bacteria and Beyond. Cancer Cell.

[126] Guo S., Chen J., Chen F., Zeng Q., Liu W.L., Zhang G. (2021). Exosomes derived from Fusobacterium nucleatum-infected colorectal cancer cells facilitate tumour metastasis by selectively carrying miR-1246/92b-3p/27a-3p and CXCL16. Gut.

[127] Domenis R., Cifu A., Marino D., Fabris M., Niazi K.R., Soon-Shiong P., Curcio F. (2019). Toll-like Receptor-4 Activation Boosts the Immunosuppressive Properties of Tumor Cells-derived Exosomes. Sci. Rep..

[128] Channon L.M., Tyma V.M., Xu Z., Greening D.W., Wilson J.S., Perera C.J., Apte M.V. (2022). Small extracellular vesicles (exosomes) and their cargo in pancreatic cancer: Key roles in the hallmarks of cancer. Biochim. Biophys. Acta. Rev. Cancer.

[129] Liu K., Yang X., Zeng M., Yuan Y., Sun J., He P., Sun J., Xie Q., Chang X., Zhang S. (2021). The Role of Fecal Fusobacterium nucleatum and pks(+) Escherichia coli as Early Diagnostic Markers of Colorectal Cancer. Dis. Markers.

[130] Bullman S., Pedamallu C.S., Sicinska E., Clancy T.E., Zhang X., Cai D., Neuberg D., Huang K., Guevara F., Nelson T. (2017). Analysis of Fusobacterium persistence and antibiotic response in colorectal cancer. Science.

[131] Kunzmann A.T., Proenca M.A., Jordao H.W., Jiraskova K., Schneiderova M., Levy M., Liska V., Buchler T., Vodickova L., Vymetalkova V. (2019). Fusobacterium nucleatum tumor DNA levels are associated with survival in colorectal cancer patients. Eur. J. Clin. Microbiol. Infect. Dis..

[132] Zhao K., Hu Y. (2020). Microbiome harbored within tumors: A new chance to revisit our understanding of cancer pathogenesis and treatment. Signal Transduct. Target Ther..

[133] Michaud D.S., Joshipura K., Giovannucci E., Fuchs C.S. (2007). A prospective study of periodontal disease and pancreatic cancer in US male health professionals. J. Natl. Cancer. Inst..

[134] Mima K., Nishihara R., Qian Z.R., Cao Y., Sukawa Y., Nowak J.A., Yang J., Dou R., Masugi Y., Song M. (2016). Fusobacterium nucleatum in colorectal carcinoma tissue and patient prognosis. Gut.

[135] Lee J.B., Kim K.A., Cho H.Y., Kim D., Kim W.K., Yong D., Lee H., Yoon S.S., Han D.H., Han Y.D. (2021). Association between Fusobacterium nucleatum and patient prognosis in metastatic colon cancer. Sci. Rep..

[136] Ai L., Xu A., Xu J. (2020). Roles of PD-1/PD-L1 Pathway: Signaling, Cancer, and Beyond. Adv. Exp. Med. Biol..

[137] Jiang S.S., Xie Y.L., Xiao X.Y., Kang Z.R., Lin X.L., Zhang L., Li C.S., Qian Y., Xu P.P., Leng X.X. (2023). Fusobacterium nucleatum-derived succinic acid induces tumor resistance to immunotherapy in colorectal cancer. Cell Host Microbe.

[138] LaCourse K.D., Zepeda-Rivera M., Kempchinsky A.G., Baryiames A., Minot S.S., Johnston C.D., Bullman S. (2022). The cancer chemotherapeutic 5-fluorouracil is a potent Fusobacterium nucleatum inhibitor and its activity is modified by intratumoral microbiota. Cell Rep..

[139] Liang M., Liu Y., Zhang Z., Yang H., Dai N., Zhang N., Sun W., Guo Y., Kong J., Wang X. (2022). Fusobacterium nucleatum induces MDSCs enrichment via activation the NLRP3 inflammosome in ESCC cells, leading to cisplatin resistance. Ann. Med..

[140] Zhang J.W., Zhang D., Yin H.S., Zhang H., Hong K.Q., Yuan J.P., Yu B.P. (2023). Fusobacterium nucleatum promotes esophageal squamous cell carcinoma progression and chemoresistance by enhancing the secretion of chemotherapy-induced senescence-associated secretory phenotype via activation of DNA damage response pathway. Gut Microbes..

[141] Da J., Wang X., Li L., Xu Y. (2021). Fusobacterium nucleatum Promotes Cisplatin-Resistance and Migration of Oral Squamous Carcinoma Cells by Up-Regulating Wnt5a-Mediated NFATc3 Expression. Tohoku J. Exp. Med..

[142] Salem A.A., Mackenzie G.G. (2018). Pancreatic cancer: A critical review of dietary risk. Nutr. Res..

[143] Barker H.E., Paget J.T., Khan A.A., Harrington K.J. (2015). The tumour microenvironment after radiotherapy: Mechanisms of resistance and recurrence. Nat. Rev. Cancer.

[144] Goudarzi M., Mak T.D., Jacobs J.P., Moon B.H., Strawn S.J., Braun J., Brenner D.J., Fornace A.J., Li H.H. (2016). An Integrated Multi-Omic Approach to Assess Radiation Injury on the Host-Microbiome Axis. Radiat. Res..

[145] Berry S.E., Valdes A.M., Drew D.A., Asnicar F., Mazidi M., Wolf J., Capdevila J., Hadjigeorgiou G., Davies R., Al Khatib H. (2020). Human postprandial responses to food and potential for precision nutrition. Nat. Med..

[146] Gibson G.R., Scott K.P., Rastall R.A., Tuohy K.M., Hotchkiss A.T., Dubert-Ferrandon A., Gareau M.G., Murphy E.F., Saulnier D., Loh G. (2010). Dietary prebiotics: Current status and new definition. Food Sci. Technol. Bull. Funct. Foods.

[147] Englyst H.N., Macfarlane G.T. (1986). Breakdown of resistant and readily digestible starch by human gut bacteria. J. Sci. Food Agric..

[148] Zafari N., Velayati M., Fahim M., Maftouh M., Pourali G., Khazaei M., Nassiri M., Hassanian S.M., Ghayour-Mobarhan M., Ferns G.A. (2022). Role of gut bacterial and non-bacterial microbiota in alcohol-associated liver disease: Molecular mechanisms, biomarkers, and therapeutic prospective. Life Sci..

[149] Hersi F., Elgendy S.M., Al Shamma S.A., Altell R.T., Sadiek O., Omar H.A. (2022). Cancer immunotherapy resistance: The impact of microbiome-derived short-chain fatty acids and other emerging metabolites. Life Sci..

[150] Gomes S., Rodrigues A.C., Pazienza V., Preto A. (2023). Modulation of the Tumor Microenvironment by Microbiota-Derived Short-Chain Fatty Acids: Impact in Colorectal Cancer Therapy. Int. J. Mol. Sci..

[151] Shoaf K., Mulvey G.L., Armstrong G.D., Hutkins R.W. (2006). Prebiotic galactooligosaccharides reduce adherence of enteropathogenic Escherichia coli to tissue culture cells. Infect. Immun..

[152] Monteagudo-Mera A., Rastall R.A., Gibson G.R., Charalampopoulos D., Chatzifragkou A. (2019). Adhesion mechanisms mediated by probiotics and prebiotics and their potential impact on human health. Appl. Microbiol. Biotechnol..

[153] Pourali G., Kazemi D., Chadeganipour A.S., Arastonejad M., Kashani S.N., Pourali R., Maftooh M., Akbarzade H., Fiuji H., Hassanian S.M. (2024). Microbiome as a biomarker and therapeutic target in pancreatic cancer. BMC Microbiol..

[154] Chen D., Wu J., Jin D., Wang B., Cao H. (2019). Fecal microbiota transplantation in cancer management: Current status and perspectives. Int. J. Cancer.

[155] Petrof E.O., Claud E.C., Gloor G.B., Allen-Vercoe E. (2013). Microbial ecosystems therapeutics: A new paradigm in medicine?. Benef. Microbes.

[156] Li S.S., Zhu A., Benes V., Costea P.I., Hercog R., Hildebrand F., Huerta-Cepas J., Nieuwdorp M., Salojarvi J., Voigt A.Y. (2016). Durable coexistence of donor and recipient strains after fecal microbiota transplantation. Science.

[157] Yang R., Chen Z., Cai J. (2023). Fecal microbiota transplantation: Emerging applications in autoimmune diseases. J. Autoimmun..

[158] Jia F., Yu Q., Wang R., Zhao L., Yuan F., Guo H., Shen Y., He F. (2023). Optimized Antimicrobial Peptide Jelleine-I Derivative Br-J-I Inhibits Fusobacterium Nucleatum to Suppress Colorectal Cancer Progression. Int. J. Mol. Sci..

[159] Van der Merwe M., Van Niekerk G., Botha A., Engelbrecht A.M. (2021). The onco-immunological implications of Fusobacterium nucleatum in breast cancer. Immunol. Lett..

[160] Baik J.E., Li L., Shah M.A., Freedberg D.E., Jin Z., Wang T.C., Han Y.W. (2022). Circulating IgA Antibodies Against Fusobacterium nucleatum Amyloid Adhesin FadA are a Potential Biomarker for Colorectal Neoplasia. Cancer Res. Commun..

[161] Tahara T., Yamamoto E., Suzuki H., Maruyama R., Chung W., Garriga J., Jelinek J., Yamano H.O., Sugai T., An B. (2014). Fusobacterium in colonic flora and molecular features of colorectal carcinoma. Cancer Res..

[162] Dejea C.M., Fathi P., Craig J.M., Boleij A., Taddese R., Geis A.L., Wu X., DeStefano Shields C.E., Hechenbleikner E.M., Huso D.L. (2018). Patients with familial adenomatous polyposis harbor colonic biofilms containing tumorigenic bacteria. Science.

[163] Bennett J.E., Dolin R., Blaser M.J., Douglas R.G. (2015). Mandell, Douglas, and Bennett’s Principles and Practice of Infectious Diseases.

[164] Gur C., Ibrahim Y., Isaacson B., Yamin R., Abed J., Gamliel M., Enk J., Bar-On Y., Stanietsky-Kaynan N., Coppenhagen-Glazer S. (2015). Binding of the Fap2 protein of Fusobacterium nucleatum to human inhibitory receptor TIGIT protects tumors from immune cell attack. Immunity.

[165] Lusiak-Szelachowska M., Weber-Dabrowska B., Gorski A. (2020). Bacteriophages and Lysins in Biofilm Control. Virol. Sin..

[166] Goswami A., Sharma P.R., Agarwal R. (2021). Combatting intracellular pathogens using bacteriophage delivery. Crit. Rev. Microbiol..

[167] Van Belleghem J.D., Dabrowska K., Vaneechoutte M., Barr J.J., Bollyky P.L. (2018). Interactions between Bacteriophage, Bacteria, and the Mammalian Immune System. Viruses.

[168] Kabwe M., Dashper S., Tucci J. (2022). The Microbiome in Pancreatic Cancer-Implications for Diagnosis and Precision Bacteriophage Therapy for This Low Survival Disease. Front Cell Infect. Microbiol..

[169] Farrugia C., Stafford G.P., Murdoch C. (2020). Porphyromonas gingivalis Outer Membrane Vesicles Increase Vascular Permeability. J. Dent. Res..

[170] Zhang Z., Liu D., Liu S., Zhang S., Pan Y. (2020). The Role of Porphyromonas gingivalis Outer Membrane Vesicles in Periodontal Disease and Related Systemic Diseases. Front Cell Infect. Microbiol..

[171] Yamamoto S., Kinugasa H., Hirai M., Terasawa H., Yasutomi E., Oka S., Ohmori M., Yamasaki Y., Inokuchi T., Harada K. (2021). Heterogeneous distribution of Fusobacterium nucleatum in the progression of colorectal cancer. J. Gastroenterol. Hepatol..

[172] Luo Z.W., Zhu M.G., Zhang Z.Q., Ye F.J., Huang W.H., Luo X.Z. (2019). Increased expression of Ki-67 is a poor prognostic marker for colorectal cancer patients: A meta analysis. BMC Cancer.

